# Synergizing photodynamic therapy and ethanol ablation: Light‐activatable sustained‐exposure ethanol injection technology for enhanced tumor ablation

**DOI:** 10.1002/btm2.70028

**Published:** 2025-05-15

**Authors:** Chen‐Hua Ma, Jeffrey Yang, John A. Quinlan, Kathryn McNaughton, Michele L. Kaluzienski, Tessa Hauser, Matthew F. Starost, Jenna L. Mueller, Huang‐Chiao Huang

**Affiliations:** ^1^ Fischell Department of Bioengineering University of Maryland College Park Maryland USA; ^2^ Division of Veterinary Resources, Office of Research Services National Institutes of Health Bethesda Maryland USA; ^3^ Marlene & Stewart Greenebaum Comprehensive Cancer Center University of Maryland School of Medicine Baltimore Maryland USA

**Keywords:** ethanol ablation, ethyl cellulose, fluorescence‐guided therapy, intratumoral photosensitizer delivery, photodynamic therapy

## Abstract

Chemical ablative therapies offer effective alternatives for tumor treatment, particularly when surgical resection or heat‐based ablation therapies are unsuitable due to the tumor's stage, location, or extent. Photodynamic therapy (PDT), which involves delivering light‐activated, tumor‐killing photosensitizers, and percutaneous ethanol injection (PEI), which involves the direct injection of pure ethanol into tumor nodules, are two non‐heat‐based chemical ablative methods that have been proven safe with low adverse effects for unresectable tumors. We have investigated combining these two treatments using a new formulation known as BPD‐EC‐EtOH. This formulation includes three components: (1) benzoporphyrin derivative, a commonly used photosensitizer for PDT; (2) ethyl cellulose (EC), an FDA‐approved polymer that forms a gel in the water phase and enhances drug retention; and (3) pure ethanol for PEI application. Here, we demonstrated the localization of BPD and confirmed that it retains its photochemical properties within the EC‐EtOH gel in tissue‐mimicking phantoms and in swine liver tissues. We also characterized EC's ability to act as a light‐scattering agent, which effectively extends light propagation distance in both in vitro models and ex vivo porcine liver tissues, potentially overcoming the limitations of light penetration in pigmented organs. We then investigated the therapeutic effects of BPD‐EC‐EtOH using two well‐established subcutaneous animal models of hepatocellular carcinoma and pancreatic ductal adenocarcinoma, both in single‐ and multi‐cycle combination treatments, showing tumor‐killing effects. These findings highlight the potential of BPD‐EC‐EtOH as a novel therapeutic approach, effective with either single or multi‐cycle treatment sessions.

**Translational Impact Statement**

This study introduces BPD‐EC‐EtOH, a novel formulation that combines photodynamic therapy and percutaneous ethanol injection for non‐heat‐based tumor ablation. Designed to treat unresectable tumors, BPD‐EC‐EtOH demonstrates promising tumor‐killing effects both in vivo and in vitro in models of hepatocellular carcinoma and pancreatic ductal adenocarcinoma. By enhancing light penetration and drug retention, this formulation highlights the potential of BPD‐EC‐EtOH as a minimally invasive therapy, providing a new option for patients with advanced stage cancers.

## 1 INTRODUCTION

While surgical resection of tumors is at the foundation of cancer treatment, many patients are ineligible for surgery due to tumor location, extent, and/or comorbidities. Consequently, ablative therapies, which locally destroy tumors through minimally invasive approaches, have become an important alternative treatment modality for patients with unresectable tumors.^1^ For instance, hepatocellular carcinoma (HCC), a leading cause of cancer mortality worldwide, is unresectable in 80% of patients due to underlying liver damage and limited volume of the future liver remnant.^2^, ^3^ Further, resection is associated with a 70% recurrence rate and is therefore not a cure for most patients. If diagnosed early enough, HCC can be treated via percutaneous tumor ablation (i.e., tumor ablation performed through the skin typically under image guidance with ultrasound, computed tomography, or magnetic resonance imaging), which has been proven to be an effective alternative to surgical excision in several settings. Radiofrequency ablation (RFA), which generates hyperthermic damage through needle electrodes, has been widely applied for treatment of HCC.^4^ However, RFA is not recommended for treatment of tumors near intestinal loops or large blood vessels, due to the risk of thermal damage to normal tissue or the heat‐sink effect of flowing blood, making tumor tissue adjacent to it less susceptible to thermal damage.^5^, ^6^ A second example of cancer that could benefit from alternative ablative therapies is pancreatic ductal adenocarcinoma (PDAC). Only 10% of PDAC patients have tumors that could potentially be surgically resected with curative intent.^7^ Thus, the majority of PDAC patients have locally advanced and unresectable pancreatic cancer due to the invasive involvement of adjacent structures, such as nearby arteries and veins.^8^ Patients who are not candidates for surgical exploration could benefit from less invasive percutaneous approaches, such as RFA.^9^ However, large treatment fields can lead to high toxicity rates,^10^ and it is challenging to avoid bowel structures surrounding nodal areas.^11^ The heat sink effect of RFA remains a challenge in PDAC.^12^ This highlights a need for alternative forms of ablation that are not based on thermal damage.

An alternative therapy for treating unresectable tumors is photodynamic therapy (PDT). PDT employs a light‐activable photosensitizer, which, when exposed to light of a specific wavelength, creates reactive oxygen species (ROS) that initiate cell death.^13^ Traditionally, photosensitizers have been delivered via intravenous, topical, or oral routes of administration, and, for superficial lesions, light is applied directly to the skin surface using laser diodes or light‐emitting diodes. In recent years, other photosensitizer administration routes, such as intraperitoneal,^14^ intra‐arterial,^15^, ^16^, ^17^ and intratumoral injections,^18^ have gained traction for their potential in treating more advanced diseases and reducing off‐target toxicities. PDT has been used to treat various types of cancer, including HCC,^19^, ^20^ cholangiocarcinoma,^21^, ^22^, ^23^, ^24^, ^25^, ^26^ PDAC,^27^, ^28^, ^29^, ^30^ brain cancer,^31^, ^32^, ^33^, ^34^, ^35^ esophageal cancer,^36^, ^37^, ^38^, ^39^ breast cancer,^40^, ^41^, ^42^ colorectal liver metastases,^43^, ^44^, ^45^ and others.^46^, ^47^ Clinical outcomes have revealed that PDT is a promising treatment for those patients who are not eligible for heated‐based ablations. Its safety and efficacy have been evaluated in patients with bile duct invasion of unresectable HCC in Korea, showing that biliary drainage and jaundice improved, with no additional complications from photosensitizers.^48^ PDT has also been used in several PDAC clinical trials. In a phase I study, most patients showed increased pancreatic tumor necrosis after endoscopic ultrasound‐guided PDT (EUS‐PDT), confirming its efficacy with a low adverse event profile.^29^ Additionally, PDT has been used for downstaging PDAC patients and converting nonsurgical candidates to surgical candidates.^27^, ^29^, ^49^ Despite encouraging initial results, obstacles remain for the clinical translation of PDT for solid tumor treatment. The accumulation of photosensitizers in normal tissues after intravenous administration can potentially lead to skin phototoxicity and abnormal liver function. Adverse events may include exaggerated sunburn, erythema, edema, vesiculation, eczematous itching dermatitis, hyperpigmentation, photo‐onycholysis, and pseudoporphyria.^50^ Williams et al. have also demonstrated that protoporphyrin accumulation may affect liver detoxification function in rats.^51^ Additionally, poor penetration of red light in pigmented tissues (<1 cm),^52^ such as liver, reduces PDT efficacy.^53^ Finally, PDT has traditionally proved most useful in concert with other treatment modalities,^27^, ^54^, ^55^, ^56^ and further innovation into combination treatments is warranted.

Here we combined PDT with a new form of percutaneous ethanol injection (PEI) to overcome these limitations. PEI serves as an established and standard treatment for small unresectable tumors, specifically those with dimensions up to 3 cm in diameter.^57^, ^58^ PEI is particularly valuable in cases where thermal ablation carries an increased risk due to the tumor's proximity to major blood vessels. However, its effectiveness is limited due to leakage away from the injection site and poor distribution of ethanol within the tumor, which can cause off‐target side effects and reduce efficacy.^59^ This study introduces a novel strategy to overcome the limitations of both PDT and PEI by using ethyl cellulose (EC) polymer to improve intratumoral co‐delivery of ethanol and a photosensitizer. EC undergoes a sol–gel phase transition upon contact with an aqueous environment in the tumor.^60^, ^61^ By adding EC, the gelated form of the polymer can help localize within the tumor, thereby limiting off‐target toxicity and enhancing efficacy.^62^

To combine the benefits of PDT and PEI for unresectable tumors, we developed BPD‐EC‐EtOH, which is a combination of three components: (i) *benzoporphyrin derivative* (BPD), a clinically used photosensitizer for PDT, (ii) the EC polymer, serving to diminish leakage and function as a light‐scattering agent, and (iii) pure ethanol (EtOH), employed as a solvent and used to facilitate ethanol ablation. In this study, we first evaluated the colocalization of BPD within the EC gel via fluorescence imaging to confirm whether BPD maintained its photochemical properties in our formulation. We also verified whether imaging conditions may lead to photobleaching of BPD‐EC‐EtOH within ex vivo swine liver tissues and in vivo subcutaneous mouse tumors. We also characterized EC's ability to extend light propagation distance in both in vitro models and ex vivo porcine liver tissues. We then investigated the tumor‐killing effects of BPD‐EC‐EtOH using two extensively studied HCC and PDAC animal models, both in single‐ and multi‐cycle combination treatments, highlighting the potential of BPD‐EC‐EtOH as a novel therapeutic approach with either single or multiple treatment sessions.

## 2 MATERIALS AND METHODS

### 2.1 BPD‐EC‐EtOH solution preparation

**FIGURE 1:**
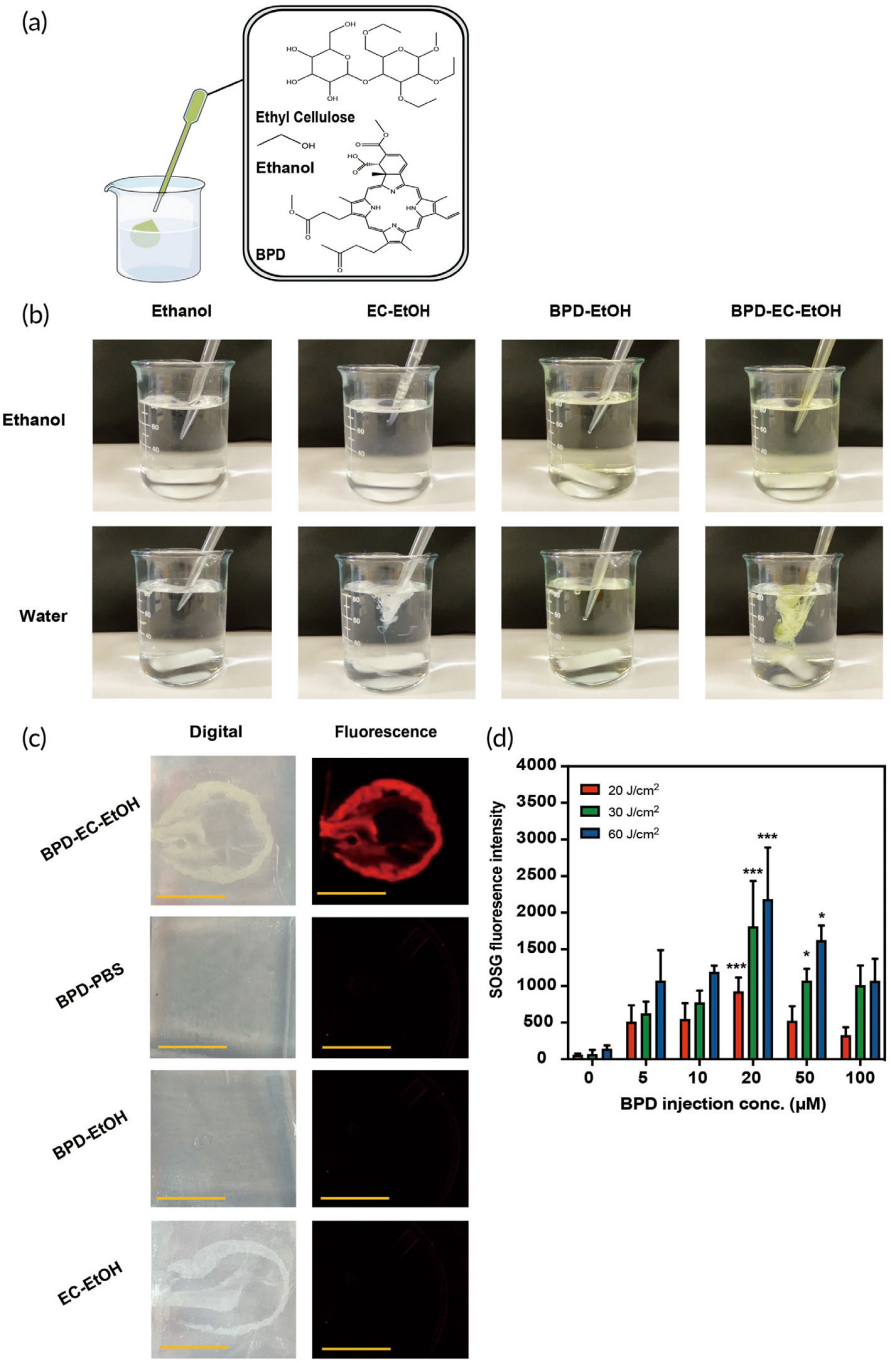
(a) Chemical structures of photosensitizer BPD, EC dissolved in ethanol (EtOH). (b) Representative image showing the formation of a BPD‐EC‐EtOH depot after a mixture of the BPD and EC dissolved in EtOH is added to water. (c) Formation of BPD‐EC‐EtOH gel depots in agar‐based tissue‐mimicking phantoms (scale bar = 10 mm). Upon red light (690 nm) activation of BPD‐EC‐EtOH, the fluorescence signal generated from BPD can be used for imaging of the depot. (d) SOSG assay revealed that BPD‐EC‐EtOH depot can be light (690 nm) activated to generate cytotoxic singlet oxygen molecules (^1^O_2_) in a BPD concentration and light dose‐dependent manner (*N* = 5, *p < 0.05; ***p < 0.001). BPD, benzoporphyrin derivative; EC, ethyl cellulose; SOSG, singlet oxygen sensor green.

A 6% mixture (weight:volume) of EC (Sigma Aldrich, St. Louis, MO) in EtOH (200 proof, Koptec, King of Prussia, PA) was prepared by stirring at room temperature.^61^ Then, 0–200 μM of BPD (US pharmacopeia, Rockville, MD) was dissolved into the EC‐EtOH solution to form a green solution (Figure 1a). The selection of this concentration range was informed by a previous study that demonstrated the existence of a plateau in effectiveness within this span.^63^ BPD concentrations were verified through measurement of its absorbance using ultraviolet–visible spectroscopy (Ex/Em: 435/685 nm), and subsequently quantified utilizing Beer's Law calculations.^64^

### 2.2 Tissue‐mimicking mechanical phantom preparation

To confirm the imageability of BPD‐EC‐EtOH in vitro, agarose‐based mechanical phantoms were used.^61^ They were composed of 1% agarose (weight:volume, UltraPure Agarose, Invitrogen, Carlsbad, CA), which was stirred into deionized water over a hot plate until a clear solution was obtained. The solution was distributed into 20‐dram polystyrene containers (Fisher Scientific, Hampton, NH) and allowed to cool at 4°C for 24 h to solidify.

### 2.3 Singlet oxygen yield detection in BPD‐EC‐EtOH

To evaluate the photoactivity of BPD‐EC‐EtOH, 5 μM of Singlet Oxygen Sensor Green (SOSG; Invitrogen, Carlsbad, CA) was added to the BPD‐EC‐EtOH solution, as described above. The formulation was then injected into an agarose phantom for further quantification of singlet oxygen. The configuration for performing injections into the mechanical phantoms is illustrated in our previous paper.^63^ All stages of solution preparation and experimentation were conducted under conditions with minimal ambient light exposure to reduce photo‐activation of both BPD and SOSG. After frontal cross‐sectional area images depicting the distribution of BPD‐EC‐EtOH within, the phantoms were captured by a fluorescence microscope with a 685‐nm laser diode (HL6750MG, Thorlabs, Newton, NJ) at 10 mW and 15 ms of exposure time, and a 6.4‐mm diameter biopsy punch was used to obtain five 5‐mm‐thick biopsy cores of the phantom. The specimens were placed in a 96‐well plate and BPD photobleaching and singlet oxygen yield in each biopsy were measured at 13 points (Figure S1). A microplate reader (BioTek) was used to acquire BPD fluorescence intensity (Ex/Em: 435/685 nm) or SOSG fluorescence signal (Ex/Em: 505/525 nm) before and after light irradiation. BPD photobleaching rate was defined as [pre‐irradiance signal at 685 nm − post‐irradiance signal at 685 nm]/pre‐irradiance signal at 685 nm, which was gathered from the microplate. Retention of BPD‐EC‐EtOH, BPD‐PBS, and BPD‐EtOH in phantom was determined (Figure S2A).

### 2.4 BPD‐EC‐EtOH detection and photobleaching in swine liver

To further evaluate the photochemical properties of BPD‐EC‐EtOH in ex vivo conditions, fresh swine liver tissue was used due to its similar size and morphology to that of human liver.^65^ Fresh swine liver was acquired from Animal Biotech Industries, Inc. 1 day before experimentation. A 3″ × 4″ × 2″ block of tissue was cut and injected with 300 μL of BPD solution using a 3‐mL syringe affixed with a 27‐gauge needle. The injection rate was held constant at 30 mL/h, and the injection depth was 13 mm. These parameters were selected based on a previous study.^60^ Retention of BPD‐EC‐EtOH, BPD‐PBS, and BPD‐EtOH in swine tissue was determined (Figure S2B). Five minutes after injection, the tissue was removed from the platform and imaged using a reflection fluorescence microscope with a 685 nm laser diode (HL6750MG, Thorlabs, Newton, NJ) at a power of 10 mW and 15 ms. The light from the laser was then collimated and projected onto the tissue. Subsequently, the light was passed through a 735 nm filter (FF01‐735/28‐25 Semrock, West Henrietta, NY) to capture the light emitted by BPD. To determine the photobleaching of each swine liver cube, a medical laser system ML7710 (Modulight, Inc., Finland) was used. Thirty minutes after injection, two 1‐cm cylindrical light‐diffusing fibers (RD10, Medlight, Finland) were placed via two 14‐gauge catheters with 5 mm separation between the two fibers. Fluorescence excitation was performed with one fiber (445 or 689 nm, 50 mW/cm), and the fluorescence signal (Em: 640–740 nm) was collected using the other (Integration time: 400 ms).

### 2.5 Light propagation in vitro and swine liver

To assess the light propagation properties of EC‐EtOH, different percentages of EC‐EtOH (0%–12%), water, and Intralipid® (Clinolipid 20%, Baxter healthcare, IL) were evaluated and compared. Intralipid® is a widely used lipid emulsion and light propagation agent that has been utilized in many clinical studies^66^, ^67^, ^68^ (Figure 3a). The solutions were first drawn up into a 30‐cm serological pipette to coat the inner walls; then, the pipette was filled with deionized water. Next, the tip of a fiber optic strand (RD10, Medlight, Finland) was inserted through the open end of the pipette. A power meter and probe (HL6750 MG, Thorlabs, Newton, NJ) were used to determine the power rating at the initial sensor position (in 10 μW/cm^2^), and the sensor was moved up in increments of 1 cm, with the power rating measured at each position. A black felt sleeve covered the entirety of the pipette, though the felt sleeve was moved as needed to expose each 1‐cm interval (Figure 3b).

To evaluate light penetration within liver tissue, swine livers underwent three consecutive injections of 1 mL of BPD‐EC‐EtOH, EC‐EtOH, or pure ethanol solutions separated at intervals of 1 cm. Injections were performed using a 1 mL syringe affixed to a 27‐gauge needle and attached to a syringe pump (Harvard Apparatus, MA). The material was injected at a constant injection rate of 30 mL/h and at an injection depth of 13 mm. Thirty minutes after injection, two 1‐cm cylindrical light diffusing fibers (RD10, Medlight, Finland) were placed within the tissue at a depth of 13 mm via two 14‐gauge catheters with 5 mm initial separation between the two fibers (Figure 3d). Fluorescence excitation was performed with one fiber (445 or 689 nm, 50 mW/cm), and the fluorescence signal (Em: 640–740 nm) was collected using the other (Integration time: 400 ms). The fluorescence signal was processed by a medical laser system (ML7710, Modulight, Inc., Finland) which was able to simultaneously capture multispectral fluorescence emission. The distance between the two fibers progressively increased until the fluorescence signal (with background subtraction) dropped below 100 relative fluorescence units (RFU).

### 2.6 Cell viability assay

Human PDAC cancer cell line, MIA PaCa‐2,^69^ and Human HCC cell line, HepG2, were purchased from the American Type Culture Collection. All cells were authenticated prior to receipt. These two cell lines were selected because they are widely used as representative PDAC and HCC studies. Cells were seeded at a concentration of 2 × 10^4^ HepG2 cells/well or 5 × 10^4^ MIA PaCa‐2 cells/well in 100 μL culture medium containing 10% fetal bovine serum and 100 U/mL penicillin/streptomycin in a clear‐bottom black‐walled 96‐well plate (Thermo Scientific, MA). Twenty‐four hours after plating, cells were incubated for 90 minutes in 0.2 μM BPD, and cells were exposed to light (bottom‐up irradiation, 690 nm, 50 mW/cm^2^, 0.1–5.0 J/cm^2^). Afterwards, the medium with BPD was removed and replaced with medium containing EtOH (0%–0.7%), and the cells were incubated for another 24 h. The medium containing EtOH was then removed; 200 μL of 3‐(4,5‐dimethylthiazol‐2‐yl)‐2,5‐diphenyl‐2*H*‐tetrazolium bromide (MTT) labeling reagent (final concentration 0.5 mg/mL) was added to each well, and plates were incubated for 1 h. Finally, 200 μL dimethyl sulfoxide (DMSO) was added to each well, the plate was allowed to stand for 15 min, and the absorbance at 580 nm was measured using a microplate reader (Synergy Neo2, BioTek, CA). The combination index (CI) is defined as CI = (half‐maximal inhibitory concentration, IC50 of EtOH in combination test/IC50 of EtOH in single test) + (IC50 of BPD + light in combination test/IC50 of BPD + light in single test). This combination index equation defines the interaction between the drugs as synergism (CI <1), additive (CI = 1), or antagonism (CI >1).

### 2.7 In vivo animal study

Male nude mice (strain: 007850, 5–6 weeks old, Jackson Laboratory) were injected subcutaneously with 5 × 10^6^ human MIA PaCa‐2 pancreatic cancer or HepG2 hepatocarcinoma cells suspended in 50 μL of phosphate‐buffered saline (PBS) plus 50 μL of Matrigel. The mice were randomized into four groups: (1) no treatment, (2) EC‐EtOH, (3) PDT (BPD + light) (690 nm, 60 J/cm^2^), and (4) BPD‐EC‐EtOH + light (690 nm, 60 J/cm^2^). Tumor size and body weight were measured every 2 days. Tumor volume was calculated using the formula volume = height × width × length × pi/6. For single‐dose treatment, once average tumors achieved a size of 60 or 250 mm^3^, depending on the study, 20 μM of BPD with 6% EC‐EtOH (60 μL) was injected intratumorally. After intratumoral injection of BPD‐EC‐EtOH or control solutions (including 20 μM BPD in PBS and 6% EC‐EtOH), the in vivo imaging system (IVIS Spectrum, Perkin Elmer, MA) was used to detect fluorescence (Figure 5a). After 90 min, the tumor was treated with a laser at 690 nm, 100 mW/cm^2^ and 60 J/cm^2^. Monitoring of tumor size and body weight was continued, and mice were euthanized after achieving a largest tumor diameter >20 mm or body weight loss >20%. For multicycle treatments, tumors were treated either when the tumor reached 100 mm^3^ or 7 days after the previous treatment, whichever was sooner.

### 2.8 Histopathology assessment of tumors

Once the mice were euthanized, the tumors were excised and snap frozen before being sectioned into 10‐μm slices with a cryostat (CM1950, Leica Microsystems, Buffalo Grove, IL). Alternating slices were stained with hematoxylin and eosin (H&E).^63^ The stained slices were then imaged with an inverted microscope (DMi8, Leica Microsystems) at a magnification of 10×. All histopathological interpretations were performed by a board certified veterinary pathologist.

### 2.9 Statistical analysis

All experiments were carried out at least in triplicate, and all statistical analyses were performed using GraphPad Prism software (GraphPad Software, San Diego, CA). Results are shown as mean ± SEM. One‐way ANOVA statistical tests and appropriate post hoc analyses were applied to avoid type I error. Unless otherwise noted, a significance level of *p* = 0.05 was applied for rejection of the null hypothesis in all analyses.

### 2.10 Ethics Statement

All animal studies were carried out according to the protocol approved by the Institutional Animal Care and Use Committee (IACUC) at the University of Maryland, College Park (Protocol R‐MAR‐22‐16).

## 3 RESULTS

### 3.1 BPD is localized within the EC gel and maintains its photochemical properties

Both BPD‐EC‐EtOH and EC‐EtOH groups immediately formed a gel in the presence of water (Figure 1b), while the ethanol and BPD‐EtOH groups readily dissolved in water. All groups were soluble in ethanol and readily dissolved in the alcohol solvent. BPD‐EC‐EtOH also formed a gel in the phantoms, with EC and BPD outlining the contours of the gel when imaged via brightfield and fluorescence microscopy, respectively (Figure 1c). EC‐EtOH also formed a depot in the phantoms but did not produce any detectable signal under fluorescence imaging. In contrast, BPD‐EtOH and BPD‐PBS failed to form a depot in phantoms due to significant leakage from the injection site and only generated a weak fluorescence signal. Regarding the photoactivity of BPD‐EC‐EtOH, there was a proportional increase in SOSG fluorescence with BPD concentration, with the peak SOSG value (525 nm; 2179 RFU) observed at 20 μM BPD with 6% EC‐EtOH under a 60 J/cm^2^ light dose (Figure 1d). The SOSG readings then plateaued at 50 and 100 μM BPD, indicating 20 μM as the minimal optimal BPD concentration for generating singlet oxygen in BPD‐EC‐EtOH.

### 3.2 BPD‐EC‐EtOH can be photoactivated in swine liver tissues

**FIGURE 2:**
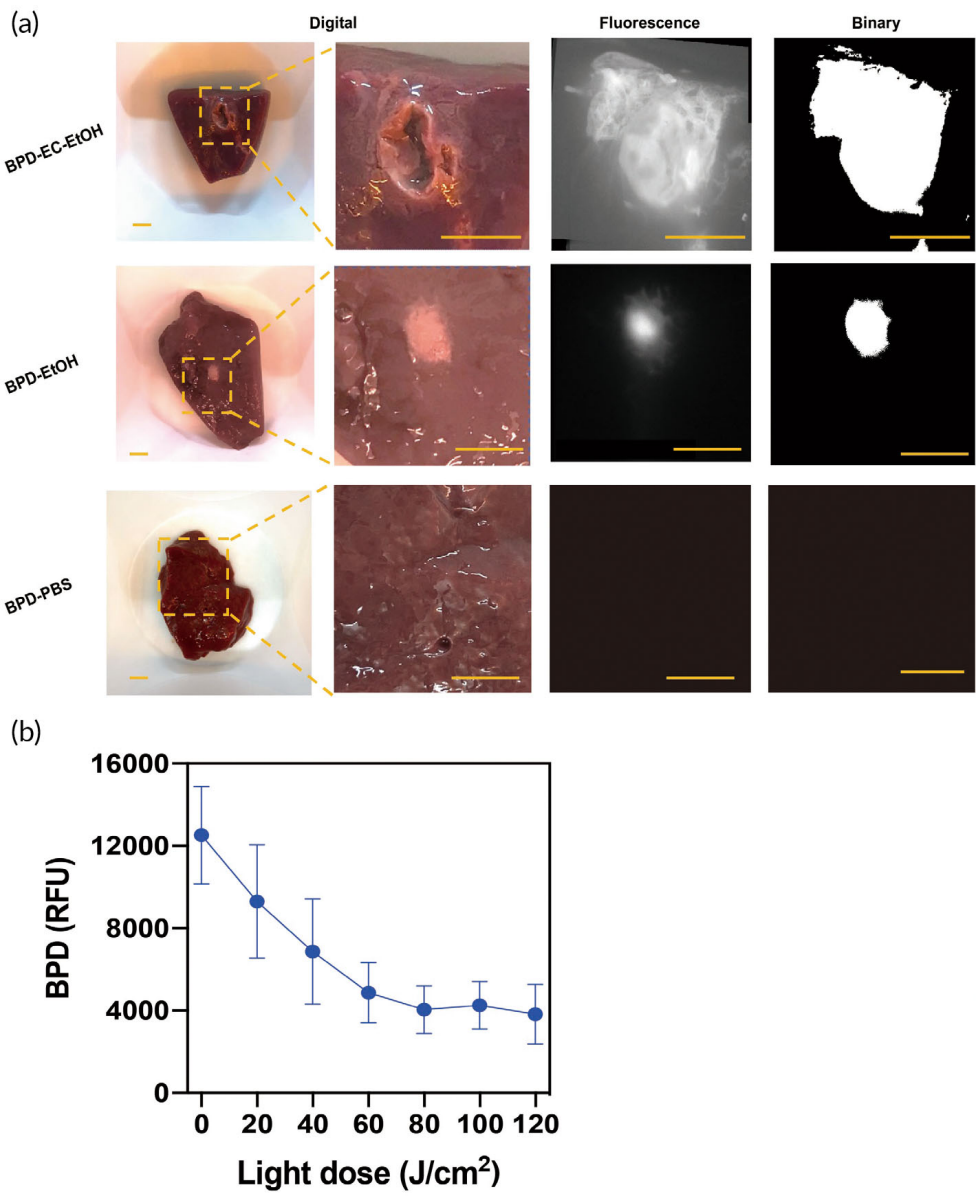
(a) Representative images showing BPD‐EC‐EtOH gel depot formation and fluorescence signal in swine liver tissue. A well‐dispersed depot and BPD fluorescence is observable in the BPD‐EC‐EtOH group, and some BPD is visible in the BPD‐EtOH group, but no fluorescent signal is visible in the BPD‐PBS group (scale bar = 1 cm). (b) In liver tissue, the singlet oxygen produced upon red light (690 nm, 50 mW/cm) activation of BPD‐EC‐EtOH resulted in BPD photobleaching, reduces BPD's fluorescence intensity (*N* = 5). BPD, benzoporphyrin derivative; EC, ethyl cellulose; PBS, phosphate‐buffered saline.

BPD‐EC‐EtOH and BPD‐EtOH injections in swine liver exhibited a white ablation zone on the liver surface (Figure 2a), but only BPD‐EC‐EtOH formed a gel depot and generated a larger distribution area. Red light irradiation of BPD‐EC‐EtOH up to 120 J/cm^2^ resulted in over 80% of the BPD undergoing photobleaching (Figure 2b). Photobleaching, described as the decay of BPD fluorescence intensity caused by light activation, is defined as [(Fluorescence intensity before light activation − Fluorescence intensity after light activation)/Fluorescence intensity before light activation] × 100%. The photobleaching of BPD after light activation correlates with the amount of singlet oxygen yield and can serve as a predictor of PDT efficacy.^70^, ^71^, ^72^ Further, a linear decrease of fluorescence signal between light dose 0–60 J/cm^2^ was observed, implying a predictable PDT light dose response, which is consistent with previous findings.^63^

### 3.3 EC increased red light propagation in vitro and ex vivo (in swine liver tissues)

**FIGURE 3:**
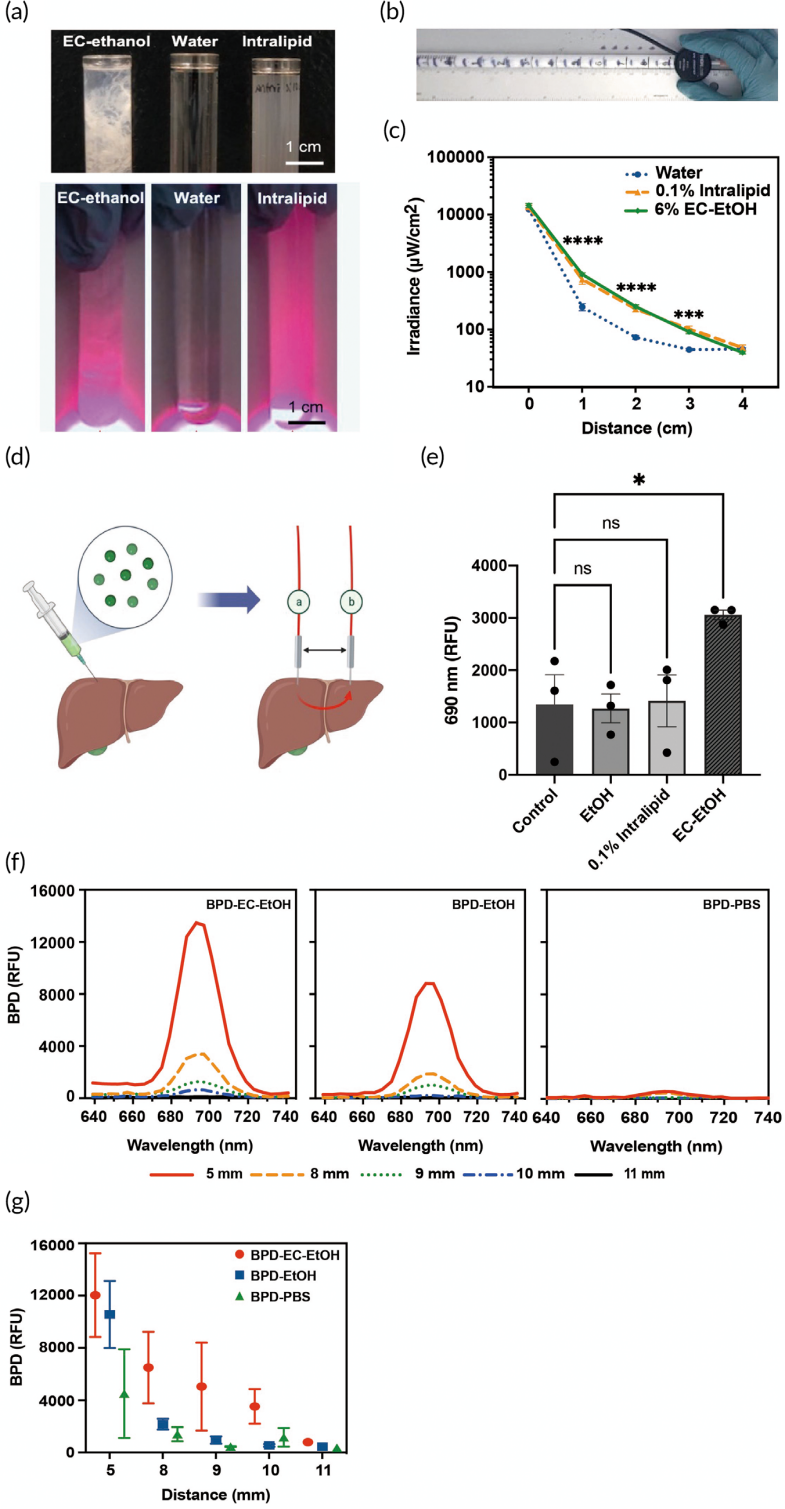
Light propagation properties of EC‐EtOH in test tubes, pipettes, and swine tissue. Light propagation (a) of EC‐EtOH in test tube. Workflow (b) and light propagation property of 6% EC‐EtOH (c) in pipettes. *p* values were marked comparing 6% EC‐EtOH and water (*N* = 3, ***p < 0.001; ****p < 0.0001). Workflow (d) and the average red‐light propagation irradiance (Ex/Em: 689/689 nm) from BPD‐EC‐EtOH, EC‐EtOH, BPD‐PBS, EtOH, and Intralipid® injected into swine liver at 15 mm distance (e). Representative fluorescence spectra (f) for detecting signal (Ex/Em: 445/640–740 nm) from BPD‐EC‐ETOH, BPD‐EtOH, and BPD‐PBS in swine liver at various depths. Average BPD fluorescence signal detected as function of laser fiber distance (g) (*N* = 3). Schematics were created with BioRender.com. BPD, benzoporphyrin derivative; EC, ethyl cellulose; PBS, phosphate‐buffered saline.

In the range of 0–4 cm from the light source, EC‐EtOH (6%) solution exhibited comparable drops in irradiance with that of the 0.1% Intralipid® positive control (Figure 3c), corresponding to similar light propagative abilities. Specifically, the EC‐EtOH gel delivered 3.68‐fold (*p* < 0.0001), 3.47‐fold (*p* < 0.0001), and 2.04‐fold (*p* < 0.001) irradiance compared to water at distances of 1, 2, and 3 cm, while 0.1% Intralipid® delivered 3.02‐fold (*p* < 0.01), 3.14‐fold (*p* < 0.0001), and 2.32‐fold (*p* < 0.0001) irradiance compared to water, respectively. To further investigate how EC facilitates light propagation in liver tissue, the depot was examined in the swine liver using two distinct wavelengths corresponding to BPD excitation wavelengths (445 and 689 nm). The average red‐light propagation irradiance (689/689 nm) at a 15 mm distance in swine liver tissue is presented in Figure 3e. Across most materials, including BPD‐EC‐EtOH, EC‐EtOH, BPD‐PBS, and 0.1% Intralipid®, the signal reached the upper detection limit of ML7710 at a 10 mm distance (16,383 RFU) between the two laser fibers, showing no significant difference (data not shown). However, at distances of 15 and 20 mm, it was observed that 6% EC‐EtOH exhibited the highest signal detection (3058 and 897 RFU, respectively) compared to other matrices (*p* < 0.05). In contrast, BPD‐PBS only returned 1192 and 363 RFU at 15 and 20 mm, respectively, while 0.1% Intralipid® returned 1344 and 756 RFU at 15 and 20 mm, respectively. This suggests that EC‐EtOH contributes to better red‐light propagation distance in swine liver tissue up to 20 mm when compared to 0.1% Intralipid®, PBS, and EtOH. Representative and average BPD fluorescence spectra (Ex/Em: 445 nm/640–740 nm) at different depths in liver tissue are illustrated in Figure 3f,g, respectively. BPD‐EC‐EtOH and BPD‐EtOH exhibited comparable light propagation capabilities, reaching 12,032 and 10,559 RFU, respectively, when the fiber distance was set to 5 mm. Both formulations displayed enhanced fluorescence signal delivery compared to BPD‐PBS, which yielded an average of 4509 RFU at the same 5 mm distance. When assessing fiber distances ranging from 5 to 11 mm, BPD‐EC‐EtOH outperformed BPD‐EtOH in terms of alleviating the light irradiance decay. Specifically, at distances of 8, 9, and 10 mm, BPD‐EC‐EtOH demonstrated higher fluorescence signals at 6499, 5042, and 3542 RFU, respectively, which were 54%, 42%, and 29% of its average irradiance at a 5 mm distance. In contrast, the BPD‐EtOH group registered signals of 2166, 953, and 543 RFU at equivalent distances of 8, 9, and 10 mm, which were 21%, 9%, and 5% of its average irradiance at a 5 mm distance. At fiber distances extending up to 11 mm, both BPD‐EC‐EtOH and BPD‐EtOH, along with BPD‐PBS, exhibited comparable low signal intensities of below 1000 RFU, which were 791, 453, and 367 RFU, respectively, and can be converted to 7%, 4%, and 8% of their average irradiance at a 5 mm distance.

The light propagation of other concentrations of EC‐EtOH, including 3%, 9%, and 12%, was also tested in pipettes (Figure S3). Within a 1 cm distance, the light propagation abilities followed a concentration‐dependent pattern, and all concentrations surpassed the performance of 0.1% Intralipid®. Between 1 and 3 cm distances, 3% EC‐EtOH exhibited the best light propagation ability, followed by other higher concentrations of EC‐EtOH and 0.1% Intralipid®. Considering the light propagation abilities at both short (0–1 cm) and long (1–3 cm) distances, as well as the previous investigation on the biodistribution of different concentrations of EC‐EtOH,^63^ we selected 6% EC‐EtOH as the optimal parameter for further studies.

### 3.4 BPD and ethanol combination showed synergistic anticancer effects in vitro

**FIGURE 4:**
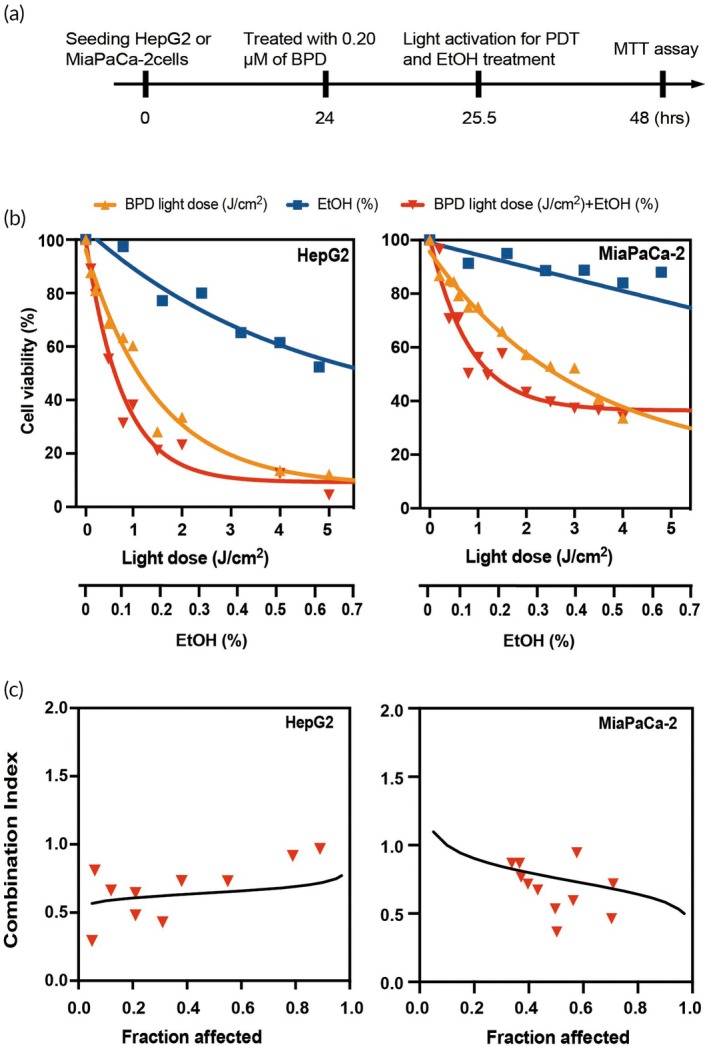
In vitro BPD and EtOH cell viability studies. (a) Assay workflow. HepG2 or MIA PaCa‐2 cells were seeded in 96‐well plates at 20,000–25,000 cells per well and allowed to grow overnight. (b) MTT cell viability assay was performed at 24 h after PDT. (c) Combination index (CI) was used to determine the degree of drug interaction or synergistic effect. BPD, benzoporphyrin derivative; MTT, 3‐(4,5‐dimethylthiazol‐2‐yl)‐2,5‐diphenyl‐2*H*‐tetrazolium bromide; PDT, photodynamic therapy.

Synergistic effects between BPD and EtOH in human hepatocarcinoma cell line, HepG2, and human pancreatic cancer cell line, MIA PaCa‐2 were evaluated (Figure 4a). The CI calculations showed that BPD and ethanol showed synergistic effects (CI <1) in both HepG2 and MIA PaCa‐2 cell lines in cell viability assays with a CI of 0.64 and 0.76, respectively (Figure 4b,c).

### 3.5 Light activation of BPD‐EC‐EtOH reduced tumor growth 14 days after treatment

**FIGURE 5:**
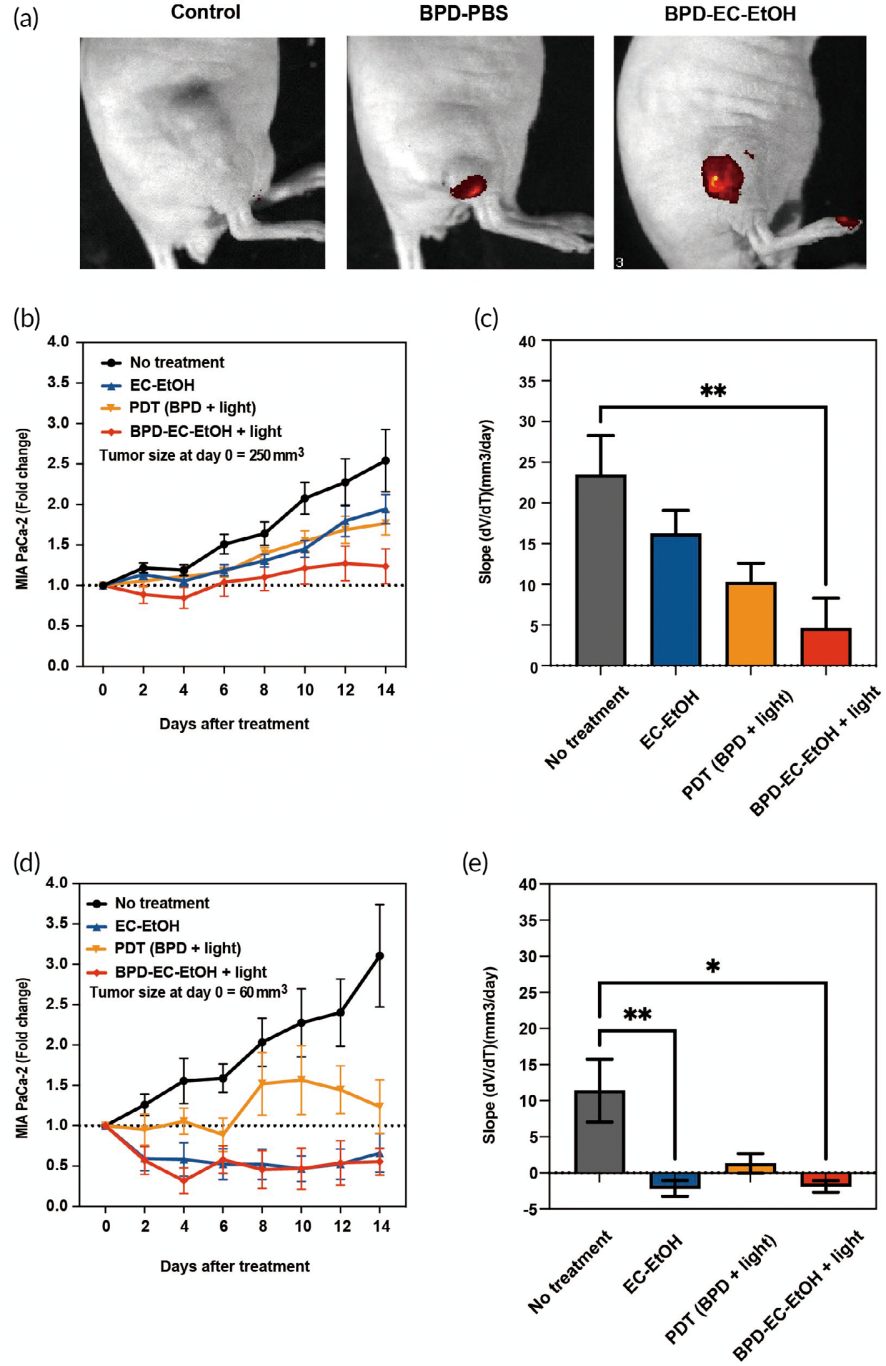
BPD‐EC‐EtOH for in vivo imaging and combination treatment. (a) Representative IVIS images displaying the effects of no treatment, EC‐EtOH, PDT (BPD + light) and BPD‐EC‐EtOH + light in the MIA PaCa‐2 tumor mouse model. (b) Tumor growth rate and (c) growth slope for tumors with an initial size of 250 mm^3^, monitored over 14 days. (d) Tumor volume fold change and (e) growth rate (volume change per day) for tumors with an initial size of 60 mm^3^, followed up for 14 days (*N* = 5). BPD, benzoporphyrin derivative; EC, ethyl cellulose; PDT, photodynamic therapy.

The combination effect of BPD‐EC‐EtOH was further tested in mice bearing MIA PaCa‐2 or HepG2 tumors. Compared to BPD alone or no treatment, a stronger BPD fluorescence signal, both in terms of area of distribution and intensity, was obtained via IVIS imaging from the BPD‐EC‐EtOH (Figure 5a) group. In larger MIA PaCa‐2 tumors (average size = 250 mm^3^), tumor volume decreased by 15% in BPD‐EC‐EtOH + light‐treated mice at 4 days posttreatment compared to the initial volume. In contrast, control tumors grew 18.9%, PDT (BPD + light)‐treated tumors grew 11.4%, and EC‐EtOH tumors grew 5.6% in the same period (Figure 5b). Fourteen days posttreatment, BPD‐EC‐EtOH + light‐treated tumors showed the best tumor control, with only a 23.7% increase in tumor volume. In contrast, PDT (BPD + light)‐treated, EC‐EtOH‐treated, and no treatment tumors grew 76.7%, 94.3%, and 154.2%, respectively. There was also a divergence in average daily tumor growth rate at 14 days posttreatment. At this time point, tumors in the no treatment group grew 23.4 mm^3^ per day, EC‐EtOH‐treated and PDT (BPD + light)‐treated tumors grew 16.2 and 10.3 mm^3^ per day, respectively. In contrast, BPD‐EC‐EtOH + light‐treated tumors showed a significantly lower (*p* = 0.003 versus control) tumor growth rate of 4.6 mm^3^ per day (Figure 5c).

For mice with smaller MIA PaCa‐2 tumors (average size = 60 mm^3^), there was a 68.2% decrease in BPD‐EC‐EtOH + light‐treated tumor volume at 4 days posttreatment, when the largest decrease in tumor volume was observed (Figure 5d). The largest decrease in tumor volume was also observed around day 4 posttreatment in the 250 mm^3^ tumors. The EC‐EtOH‐treated group also displayed a volume decrease of 41.8% compared to the initial day. Conversely, the no treatment group had a 55.5% increase in tumor volume, and the PDT (BPD + light) group exhibited a 5.5% growth in tumor volume. After a 14‐day posttreatment, both the EC‐EtOH and BPD‐EC‐EtOH + light‐treated groups exhibited similar tumor control, with volume reductions of 33.8% and 44.6%, respectively, from their initial volumes. In contrast, the no treatment and PDT (BPD + light) groups experienced tumor volume increases of 210% and 23%, respectively. Over the same posttreatment period, the no treatment and PDT (BPD + light) groups displayed average daily tumor growth rates of 11.4 mm^3^ and 1.3 mm^3^, respectively, while the EC‐EtOH and BPD‐EC‐EtOH + light‐treated groups demonstrated average daily tumor volume decreases of approximately 2.1 and 1.9 mm^3^, respectively. Both the EC‐EtOH and BPD‐EC‐EtOH + light‐treated groups showed significant differences (*p* < 0.01 and *p* = 0.011, respectively) compared to the no treatment group (Figure 5e). In mice with smaller MIA PaCa‐2 tumors (average size = 60 mm^3^), no significant difference in tumor control was observed between the EC‐EtOH and BPD‐EC‐EtOH + light‐treated groups. We believe that the challenge in observing synergistic effects of combination treatments in smaller tumors arises from several factors. First, drug penetration in smaller tumors is generally more uniform, making single‐agent treatments appear more effective. In contrast, larger tumors often exhibit poor perfusion and hypoxic regions, where one drug may fail while another compensates, enhancing the observed synergy. Second, smaller tumors tend to have a higher fraction of proliferating cells, increasing their sensitivity to single‐agent therapies. Lastly, smaller tumors display less heterogeneity, reducing the likelihood of synergy between treatments.

**FIGURE 6:**
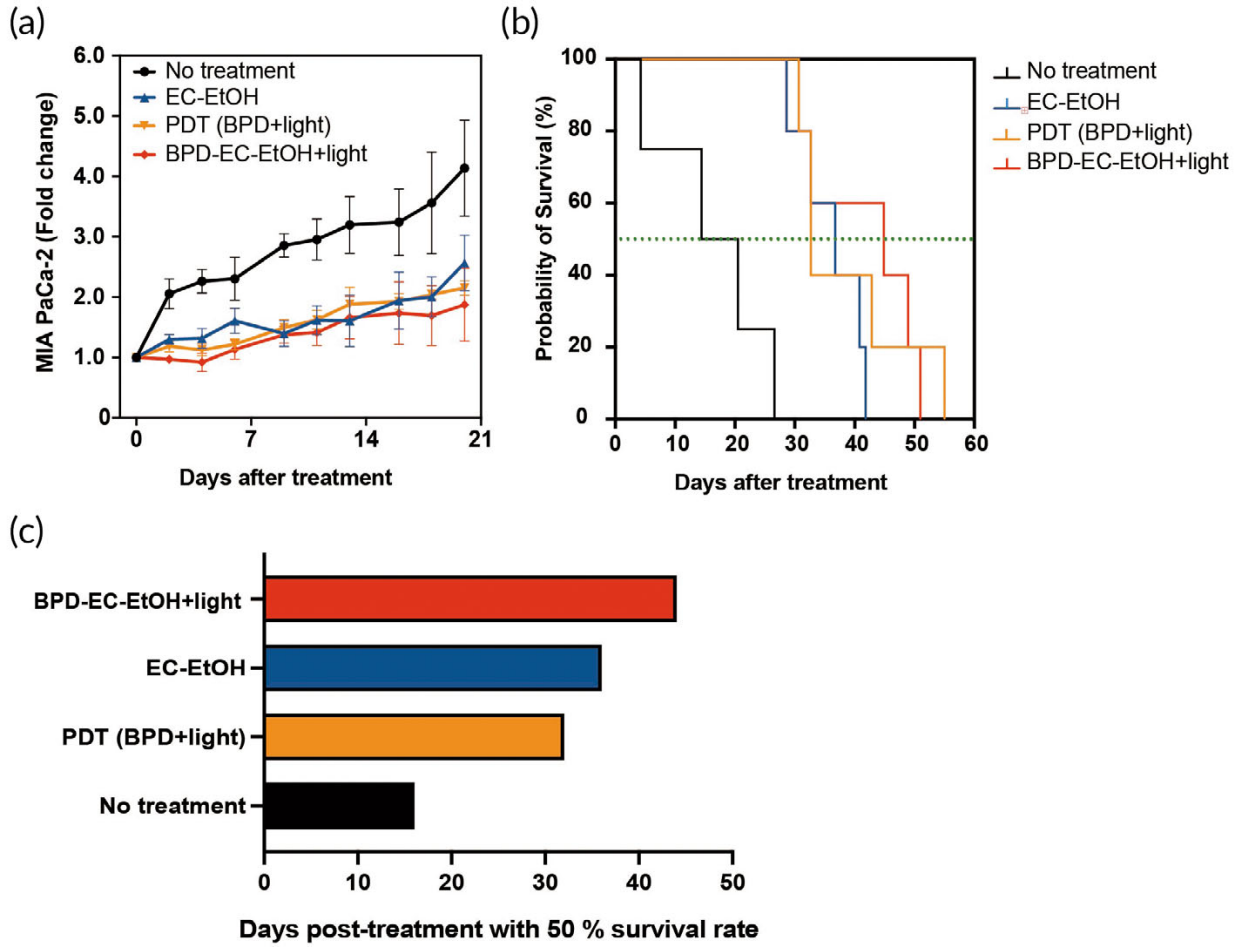
BPD‐EC‐EtOH for multicycle combination treatment in the MIA PaCa‐2 tumor mouse model. Tumors were treated up to three times with 7‐day intervals between treatments only if the tumor regrew to above 100 mm^3^ within 7 days. If the tumor did not reach this threshold, the subsequent treatment was postponed until the tumor volume reached the threshold of 100 mm^3^. (a) Average tumor volume fold change of each group, including no treatment, EC‐EtOH, PDT (BPD + light) and BPD‐EC‐EtOH + light. The one‐way analysis of variance indicated a significant difference (*p* < 0.05) between the no‐treatment group and the BPD‐EC‐EtOH + light group. (b) Survival curve, and (c) the days posttreatment when 50% of survival was observed (*N* = 5). BPD, benzoporphyrin derivative; EC, ethyl cellulose; PDT, photodynamic therapy.

**FIGURE 7:**
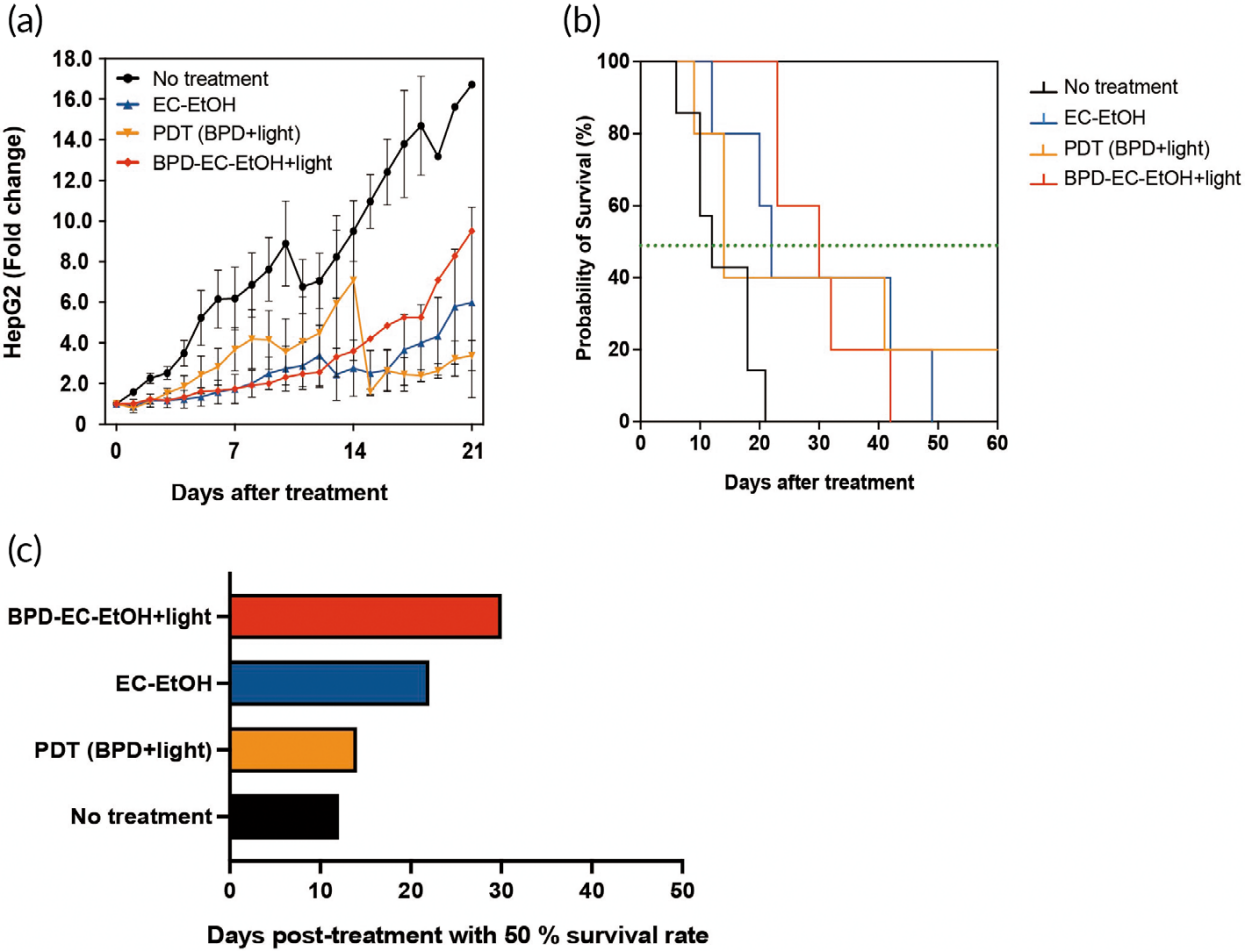
BPD‐EC‐EtOH for multicycle combination treatment in HepG2 tumor‐bearing mice with an initial tumor volume of 100 mm^3^. Tumors were treated up to three times with 7‐day intervals between treatments only if the tumor regrew to above 100 mm^3^ within 7 days. If the tumor did not reach this threshold, the subsequent treatment was postponed until the tumor volume reached the threshold of 100 mm^3^. (a) Normalized tumor size of individual animal tumor burden of no treatment, EC‐EtOH, PDT (BPD + light), and BPD‐EC‐EtOH + light groups. The one‐way analysis of variance indicated a highly significant difference (*p* < 0.0001) between the no‐treatment group and all other groups. Survival rate (b) and the days posttreatment when 50% of survival was observed (c) (*N* = 5). BPD, benzoporphyrin derivative; EC, ethyl cellulose; PDT, photodynamic therapy.

Building upon the findings of the single treatment study, we conducted a multicycle treatment scheme to enhance BPD‐EC‐EtOH + light efficacy in both HepG2 and MIA PaCa‐2 tumor‐bearing animal models, spaced 7 days apart for a total of 3 treatments. In the MIA PaCa‐2 tumor mouse model, heterogeneous outcomes were observed within all treatment groups (Figure S4). Analysis of the average tumor fold change following each treatment revealed results consistent with those of the previous single treatment study, indicating that the tumor volume in the BPD‐EC‐EtOH + light group reached a minimum volume after 4 days of each treatment in the multicycle scheme (i.e., on days 4, 11, and 18) (Figure 6a). Although the median survival days increased in the BPD‐EC‐EtOH + light group, overall survival rates did not significantly improve (Figure 6b). Median survival in the BPD‐EC‐EtOH + light group was 44 days, while the EC‐EtOH, PDT (BPD + light), and no treatment groups had median survivals of 36, 32, and 16 days, respectively (Figure 6c). Similar heterogeneous outcomes were observed in the HepG2 model (Figure S5). In the HepG2 model, BPD‐EC‐EtOH + light and EC‐EtOH had comparable anti‐tumor efficacy (Figure 7a). Although the median survival days increased in the BPD‐EC‐EtOH + light group, there was no significant improvement in overall survival rates (Figure 7b). In the HepG2 model, BPD‐EC‐EtOH + light, EC‐EtOH, PDT (BPD + light), and no treatment groups experienced median survival of 30, 22, 14, and 12 days, respectively (Figure 7c).

### 3.6 Histopathology assessment indicated BPD‐EC‐EtOH induced multiple types of necrosis

**FIGURE 8:**
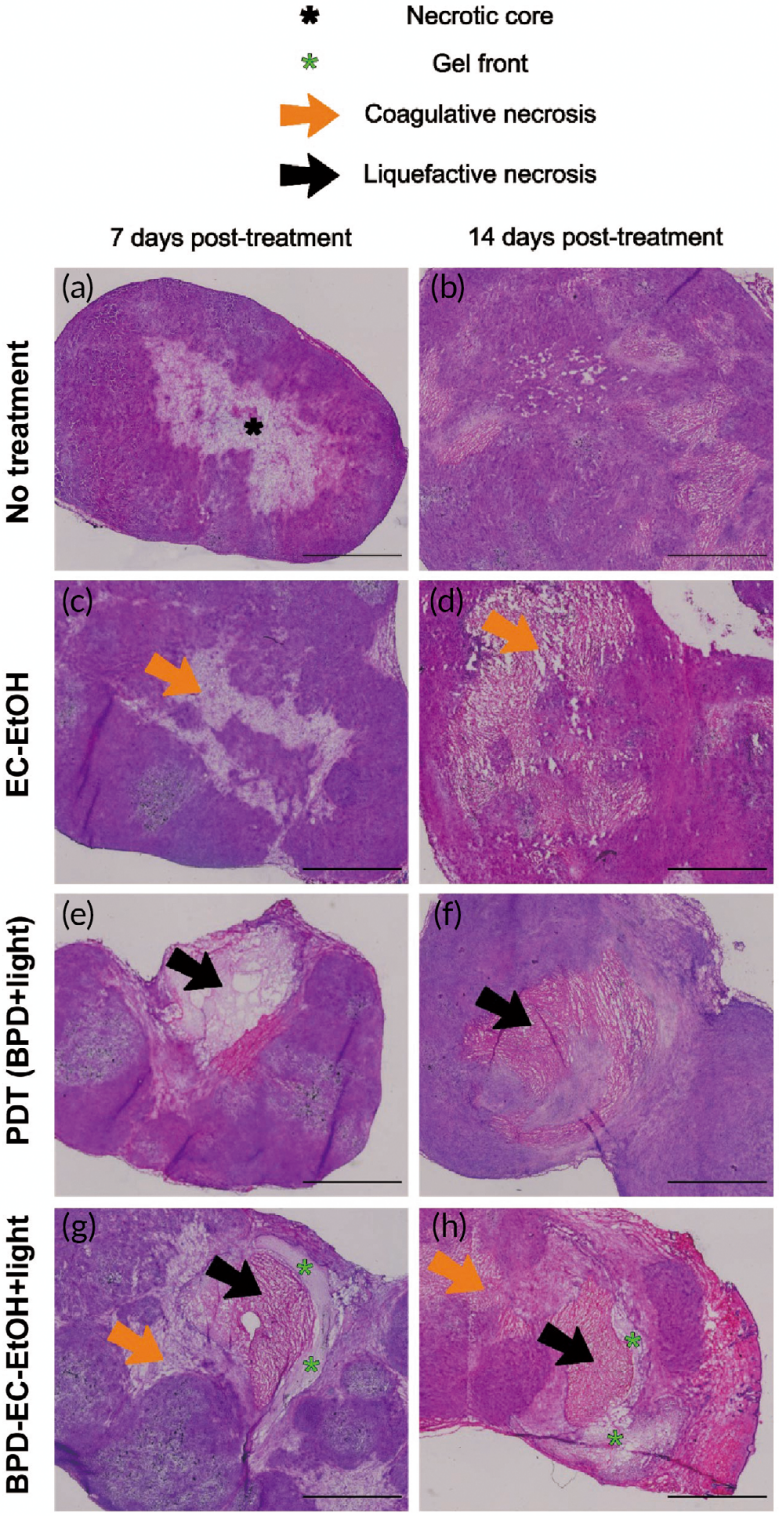
H&E pathology analysis. MIA PaCa‐2 tumors (a, c, e, g) 7 days and (b, d, f, h) 14 days posttreatment. A necrotic core is apparent in the no treatment group (a), with coagulative necrosis induced in the PDT (BPD + light) group (c, d), liquefactive necrosis induced in the EC‐EtOH group (e, f), and both types of necrosis induced in the BPD‐EC‐EtOH + light group (g, h). Scale bars = 2 cm. BPD, benzoporphyrin derivative; EC, ethyl cellulose; PDT, photodynamic therapy.

All treatment groups displayed distinct regions of necrosis that delineated the ablation treatment zone (Figure 8). The no treatment group was observed to either have a necrotic core at the center of the tumor or small, scattered pockets of necrosis (Figure 8a,b). Notably, the PDT (BPD + light) group showed signs of coagulative necrosis (Figure 8c,d), while the EC‐EtOH and BPD‐EC‐EtOH + light groups additionally showed signs of liquefactive necrosis in the center of the ablation zone (Figure 8e–h). At 7‐day post‐ablation, connective tissue was observed for the PDT (BPD + light) and BPD‐EC‐EtOH + light group (Figure 8c,g), while the EC‐EtOH group did not show signs of connective tissue deposition (Figure 8e). The gel front induced by EC was characterized by the pale, acellular regions within the ablation zone, and was prominent at the periphery of the ablation zone for the BPD‐EC‐EtOH + light treatment group (Figure 8g). At 14‐day post‐ablation, there was partial resolution of the ablation zone into viable tissue in the control and PDT (BPD + light) treatment groups (Figure 8b,d), with the latter displaying fibrovascular stroma at the margins of the ablation zone. While some connective tissue was observed in EC‐EtOH and BPD‐EC‐EtOH + light (Figure 8f,h), there remained signs of liquefactive necrosis and little connective tissue present. The gel front remained visible for BPD‐EC‐EtOH + light at all time points (Figure 8g,h).

## 4 DISCUSSION

Certain solid tumors present intricate challenges for surgical interventions due to factors such as their dimensions, contours, and placement, necessitating the exploration of alternative treatment approaches. Patients with conditions like HCC or PDAC frequently encounter scenarios where surgical procedures remain unfeasible due to vascular complexities.^73^, ^74^, ^75^, ^76^ While percutaneous thermal ablation effectively manages some solid tumors, it is not advisable for tumors situated near intestinal loops or prominent blood vessels due to the potential risk of thermal damage to healthy tissue or the influence of blood flow on temperature distribution.^1^, ^5^, ^6^, ^77^ Moreover, the procedure requires specialized magnetic resonance imaging equipment and extensive training for physicians, restricting its widespread application, especially among underserved populations.^78^, ^79^ There is an urgent need to develop novel ablative strategies to manage both currently difficult‐to‐treat tumors in well‐served areas and to offer new treatment choices for underserved areas. In response to these challenges, chemical ablative therapies have emerged as promising avenues for effectively addressing unresectable tumors.

The two chemical‐based treatment methods explored here, PDT and PEI, both exhibit minimal adverse effects and are feasible in tumors that present a higher surgical risk due to their proximity to blood vessels. PEI was extensively utilized in Europe and many Asian countries and has been recommended as the standard percutaneous treatment for early‐stage, inoperable HCC.^80^, ^81^ Furthermore, in Korea, an EUS‐guided PEI combined with a chemotherapy trial appears to be safe for the treatment and resolution of pancreatic cysts, with only mild pancreatitis and splenic vein obliteration.^82^, ^83^ The combination of photosensitizers and ethanol has been investigated exclusively in the context of antimicrobial PDT for treating periodontal and infectious diseases, such as against *Pseudomonas* aeruginosa biofilms,^84^ resistant *Staphylococcus aureus*,^85^ and other Gram‐negative pathogens,^86^ as well as for enhancing the solubility and penetration of photosensitizers into the skin.^87^ Previous studies have shown that PDT combined with ethanol is effective. To further enhance the efficacy of PDT and PEI, the primary objective of this research is to evaluate the combined use of PDT and PEI for treating locally unresectable solid tumors for the first time.

To address issues commonly associated with intratumoral drug delivery, including leakage and insufficient distribution, the incorporation of EC was introduced into the formulation of BPD‐EC‐EtOH. The addition of EC has been shown to significantly enhance the distribution and reduce leakage of the formula mixture across various materials, including tissue‐mimicking agarose phantoms, swine liver tissues, and two distinct mouse tumor models, as demonstrated in both our previous study^63^ and the current research. The retention of BPD‐EC‐EtOH at the tumor injection site has been shown to last for over 7 days under fluorescence imaging, compared to less than 24 h for the BPD‐PBS control. Moreover, only about 5% of BPD and ethanol were released from the depot after 7 days, indicating the potential for multi‐session PDT within a 7‐day period following the initial injection of BPD‐EC‐EtOH. Based on phantom injection data, we have demonstrated that an injection volume of 300 μL of BPD‐EC‐EtOH can cover an area up to 2 cm in diameter, with the injection volume‐to‐tumor volume ratio controlled between 25% and 100% in this study. Considering the light penetration distance, treatment coverage can be extended up to 3–4 cm (Figure 3c), which is sufficient to cover tumors between 3 and 6 cm in size for HCC or PDAC tumors when using a larger injection volume. This is achievable by increasing the BPD‐EC‐EtOH injection volume‐to‐tumor volume ratio to 100% or higher, as done in previous PEI clinical studies to provide a safety margin.^57^, ^88^

An additional challenge previously highlighted in PDT is the limited depth of light penetration from red‐light sources. The heightened light propagation achieved through EC‐EtOH is evident in both in vitro pipettes and ex vivo swine liver, demonstrating that our formulation's effectiveness is superior or comparable with that of Intralipid®, particularly within a depth of 15 mm, which is sufficient for treating a 3‐cm HCC tumor in its early phase. For a shorter wavelength (435 nm), BPD‐EC‐EtOH can expand the light propagation distance up to 10 mm compared to 8 mm in BPD‐PBS. This enhanced depth of penetration signifies an expanded coverage of tumor regions, suggesting that EC‐EtOH could be used as an alternative to the commonly utilized light‐scattering agent Intralipid® in clinical PDT applications. The effectiveness of PDT within tissues is largely influenced by the application and delivery of light, with extending the depth of light penetration allowing for reaching a larger treatment target area and potentially broadening the selection of photosensitizers.^89^ Another important factor affecting penetration depth is the scattering mechanisms within different matrices.^90^, ^91^ Scattering plays a vital role in reducing light intensity within the tissue.^92^ While light diffusion in scattering media is commonly viewed as undesirable in photonic applications, in specific materials, multiple light scattering allows for light trapping within the material, thereby increasing photon travel distance.^93^ Currently, a variety of polymer materials can be used to fabricate scattering media.^94^ Orelma et al. introduced one of the first optical fibers made from pure regenerated cellulose. The dimensions of the pores, which act as scattering media, or the non‐homogeneous pore size distribution, may have caused locally strong scattering, resulting in resonant feedback at specific points.^93^ We propose that the arrangement of cellulose polymer fibers in BPD‐EC‐EtOH may similarly enable incident light to undergo scattering in the tissue, ultimately reducing light decay. To further achieve low‐loss light scattering, the material must have high optical transparency and have a higher refractive index than the surrounding tissue. The refractive index of the EC‐EtOH gels within the context of liver tissue should be measured in future studies to confirm our observation.

A limitation of our in vitro combination study is the exclusion of EC. We opted not to include EC to prevent its gelation in the cell medium, as it could impede cell growth. In the absence of EC, in vitro MTT assays conducted on both MIA PaCa‐2 and HepG2 cells present compelling evidence of a synergistic effect (IC50 < 1) between ethanol and BPD + light, demonstrating their combined ability to induce cell death. Through verification of the photoactivity of BPD in EC‐EtOH within agarose phantoms, an optimized injection parameter (20 μM BPD, with 6% EC‐EtOH under a light dose of 60 J/cm^2^) was identified. These parameters were selected for subsequent in vivo studies because they generated the highest level of singlet oxygen in vitro. There are several potential mechanisms that may explain the observed synergistic effect of BPD‐based PDT and ethanol in the MTT assay. First, ethanol may facilitate the cellular uptake of BPD, potentially elevating its intracellular concentration, thus fostering more efficient cell destruction upon light irradiation. Studies have indicated that ethanol can directly interact with membrane proteins,^95^, ^96^ leading to conformational changes that affect their functions. Ethanol also indirectly influences the functions of membrane‐associated proteins such as receptors, ion channels, and enzymes by disrupting the physical structure of cell membranes and increasing membrane fluidity.^97^ For instance, ethanol exposure has been shown to enhance dopamine intracellular uptake in neuron cells, possibly due to the increased sensitivity of dopamine receptors following ethanol administration.^98^ Ethanol toxicity also affects the cytoskeletal system, as observed in ethanol‐treated astrocyte cultures where both the actin cytoskeleton and the microtubular network were disrupted and disorganized.^99^ Second, compared to water, EtOH might extend the longevity of singlet oxygen,^100^, ^101^ a ROS generated during PDT, potentially intensifying its cytotoxic impact. Lastly, BPD is known to aggregate in aqueous solution due to its hydrophobic nature,^102^, ^103^ but is soluble in organic solvents such as DMSO or DMF. Here it displays spectra characteristic of the free‐base chlorin type. In ethanol, BPD has exhibited self‐aggregation when its concentration exceeded 0.6 μM.^104^ In this study, we found that the inclusion of EC‐EtOH acted as a deterrent to the formation of BPD aggregates, consequently enhancing the drug's dispersion, bioavailability, and photoactivatability for in vivo use.

Single‐cycle in vivo application of BPD‐EC‐EtOH + light combination therapy in both pancreatic and HCC tumor‐bearing animal models shows promise. This is evident from the notable reduction in normalized tumor volume and the deceleration of tumor growth rate following treatment in comparison to the untreated group. These findings highlight the potential of the BPD‐EC‐EtOH + light combination as a potent therapeutic approach for combating tumors in vivo. In the multicycle treatment regimen, the BPD‐EC‐EtOH + light group demonstrated a longer median survival time, yet overall survival time and tumor burden control remained suboptimal compared to the monotherapy groups. Additionally, individual tumor sizes varied within each treatment group across both animal models, likely due to the heterogeneous tumor microenvironment (e.g., proximity to injection vasculature, degree and location of gel spread within the tumor, and effectiveness of top‐down light reaching the gel) among different animals. We also speculate that the minimal release of photosensitizer and ethanol (5%) from the gel depot may limit the effectiveness of multicycle PDT in vivo. In clinical settings, one major challenge of percutaneous ethanol ablation therapy is damage to normal tissue. To address this, we designed the gel to release low amounts of photosensitizer and ethanol, aiming to minimize harm to healthy tissue. However, restricted photosensitizer diffusion could leave some tumor regions untreated. We believe that optimizing the release rate of the photosensitizer, along with refining the interstitial light delivery technique, could enhance the anti‐tumor PDT effect while reducing damage to surrounding healthy tissue in vivo. While further research is needed to fully understand the efficacy of multicycle BPD‐EC‐EtOH + light treatment, it is noteworthy that multicycle treatment was safe and tolerable. To prevent tumor recurrence and enhance treatment efficacy, more research is crucial to determine the optimal drug dosage, light dosage and delivery route, treatment interval, and injection volume. One approach to consider is to increase the injection volume beyond the 60 μL used in this study, as clinical ethanol injection volumes are typically adjusted based on tumor size to ensure complete coverage. Additionally, we could utilize the fluorescent signal from the BPD photosensitizer to monitor depot biodistribution in real time. Finally, to enhance BPD diffusion from the gel, we could modify the polarity of BPD‐EC‐EtOH, thereby influencing the sol–gel transition and potentially facilitating BPD diffusion.

EC‐EtOH prolongs the retention of BPD within the tumor, as indicated in our previous work,^63^ potentially enhancing its light‐dependent and light‐independent effects. For example, BPD has the ability to inhibit the growth of human glioma in vitro without light activation.^105^, ^106^ These light‐independent effects should also be considered in our future work. Furthermore, the prolonged presence of EC‐EtOH gel within the tumor microenvironment may impede tumor healing, leading to a reduction in the rate of tumor growth. Nief et al. have demonstrated that injecting EC‐EtOH into triple‐negative breast cancer tumors significantly reduces tumor growth and triggers an immune response, including increased local tumor‐infiltrating lymphocytes, which produce an anti‐metastatic effect.^60^ On the other hand, Ghosh et al. reported on a late‐stage pancreatic cancer patient who was deemed unsuitable for surgery and began EUS‐guided PDT. After 7 months of PDT, there was a decrease in the size of the local pancreatic uncinate mass and distal pulmonary metastatic lesions. The study also noted an increase in CD8‐positive T‐cell population, tumor‐reactive T cells, and T cells responsive to PD‐1 therapy.^107^ These findings suggest that local ablative therapies, PDT and EC‐EtOH injections, have the potential to be combined with immunomodulators for cancer therapy^108^ and are capable of inducing a systemic anti‐tumor response, such as the abscopal effect. Employing immunocompetent mice in future animal studies is needed to explore the micro‐tumor environment and corresponding immune responses to cell death and the presence of BPD and EC polymer.^56^, ^109^, ^110^, ^111^

PDT and PEI are technologies known to induce necrosis in tissues, and the combination of the two generated both coagulative and liquefactive necrosis within the tumors (Figure 8). Including EC in the injectate allowed for longer retention of BPD and ethanol within the injection site, as EC is known to sequester ethanol through its gelation properties when exposed to aqueous solution. This may explain why the PDT (BPD + light) group started showing signs of connective tissue deposition while the EC‐containing groups (i.e., EC‐EtOH and BPD‐EC‐EtOH + light) did not display obvious regions of connective tissue or resolution of necrosis into viable tissue. Specifically, the BPD in the PDT (BPD + light) group did not contain a robust vehicle to stay localized within the tumor stroma, and thus was cleared out sooner than the other treatment groups and allowed a faster opportunity for the tumor to recur. Future studies can further investigate the contents of the connective tissue (e.g., collagen) through other histological stains such as Trichrome and Picrosirius Red. One notable observation is that the liquefactive necrotic region in the BPD‐EC‐EtOH + light tends to be closer to the injection site (the gel front can serve as a visual indicator of a portion of the ablation zone boundary), with the coagulative necrotic region populating the outer region of the ablation zone (Figure 8g,h). The transition zone between the two necrotic regions or between the viable tissue and ablation zone may hold more information about how tissues recover after exposure to PDT and PEI, and further studies would be required to characterize this peripheral region.

Lastly, although the localized treatment effect of BPD‐EC‐EtOH + light is notable in this study, further investigation into its application near large vessels or delivery via intra‐arterial injection^18^, ^112^ is warranted and worth considering. Intra‐arterial ethanol embolization has proven effective for treating HCC.^112^, ^113^ In China, Yang et al. evaluated the feasibility of combining transcatheter chemoembolization (TACE) for treating HCC with portal vein tumor thrombus, along with intra‐arterial ethanol embolization for the thrombus. Their results showed a threefold longer median survival in the treatment group compared to transcatheter chemoembolization alone.^113^ Ethanol induces an embolization effect by causing endothelial damage and thrombosis in the arteriolar lumen of tumor feeder vessels and tumor vasculature, resulting in tumor infarction. Considering these findings, our formulation may have potential for combined use with TACE to prolong the embolization effect and potentially downstage larger HCC in intermediate stages.^113^ Approximately 42,000 patients are diagnosed annually with unresectable HCC, and about 30,000 patients in the United States are diagnosed with PDAC each year. There is potential for translating BPD‐EC‐EtOH + light into clinical practice in the future. Intratumoral or intra‐arterial delivery of BPD‐EC‐EtOH combined with ultrasound, followed by laser fiber light activation, could be feasible in clinical settings for patients with locally advanced HCC and PDAC, potentially downgrading tumors to a resectable status.

## 5 CONCLUSIONS

In summary, this study introduces a novel combination of PDT and PEI using BPD‐EC‐EtOH + light therapy and demonstrates its efficacy in two different tumor models in vivo. These promising initial results support the use of combined PDT and PEI therapies, highlighting their potential for clinical applications. To further enhance the efficacy of BPD‐EC‐EtOH + light, several considerations are important. First, refining injection parameters, light dosages, and delivery methods is crucial to optimize conditions for potential clinical use. Utilizing real‐time fluorescence signal feedback from the ML7710 clinical laser system for adaptive light dosing during treatment could enhance photobleaching levels and tailor treatments for personalized medicine.^114^ Second, gaining deeper insights into the mechanisms underlying the synergistic effects of PDT and PEI in the tumor microenvironment is essential. Such research promises to provide valuable insights into the potential effectiveness of this treatment strategy in complex clinical scenarios.

## AUTHOR CONTRIBUTIONS

The manuscript was written through the contributions of all authors. All authors have given approval to the final version of the manuscript.

## FUNDING INFORMATION

This work is supported by the National Institutes of Health, R00CA234455 (J.L.M.) and R01CA260340 (H.H.) grants; the University of Maryland startup funds (H.H. and J.L.M.); the NCI/UMD Partnership for Integrative Cancer Research; and the Maryland Innovation Initiative (MII) award (H.H. and J.L.M.).

## CONFLICT OF INTEREST STATEMENT

The authors declare no conflicts of interest.

## Supporting information


**Figure S1.** Layout and details for the analysis of SOSG data. Following the injection of BPD‐EC‐EtOH into the phantoms, the front section of the phantoms was exposed. Subsequently, five 5‐mm‐thick biopsies were extracted using a hollow tool. These biopsies were labeled 1–5, with biopsy number 2 always placed at the injection site (A). The biopsies were then arranged accordingly in a 96‐well plate for subsequent BPD photobleaching and SOSG detection. Each biopsy was assessed at 13 specified points (B) using a plate reader.
**Figure S2.** Retention rate of BPD‐EC‐EtOH, BPD‐PBS, and BPD‐EtOH in phantom (A) and swine liver (B).
**Figure S3.** Light propagation study of 3%, 6%, 9%, 12% of EC‐EtOH in pipettes.
**Figure S4.** Normalized individual animal tumor burden for multicycle combination treatment in the MIA PaCa‐2 tumor mouse model of no treatment, EC‐EtOH, PDT (BPD + light), and BPD‐EC‐EtOH + light groups.
**Figure S5.** Normalized individual animal tumor burden for multicycle combination treatment in the HepG2 tumor mouse model of no treatment, EC‐EtOH, PDT (BPD + light), and BPD‐EC‐EtOH + light groups.

## Data Availability

The data that supports the findings of this study are available in the Supporting Information of this article.
