# Supplementary material for: Synergizing photodynamic therapy and ethanol ablation: Light‐activatable sustained‐exposure ethanol injection technology for enhanced tumor ablation
Source: Bioeng Transl Med. 2025 May 15;10(5):e70028. doi: 10.1002/btm2.70028 (PMC12478449; doi:10.1002/btm2.70028)
Supplement: Supplementary file 1 — Figure S1. Layout and details for the analysis of SOSG data. Following the injection of BPD‐EC‐EtOH into the phantoms, the front section of the phantoms was exposed. Subsequently, five 5‐mm‐thick biopsies were extracted using a hollow tool. These biopsies were labeled 1–5, with biopsy number 2 always placed at the injection site (A). The biopsies were then arranged accordingly in a 96‐well plate for subsequent BPD photobleaching and SOSG detection. Each biopsy was assessed at 13 specified points (B) using a plate reader. Figure S2. Retention rate of BPD‐EC‐EtOH, BPD‐PBS, and BPD‐EtOH in phantom (A) and swine liver (B). Figure S3. Light propagation study of 3%, 6%, 9%, 12% of EC‐EtOH in pipettes. Figure S4. Normalized individual animal tumor burden for multicycle combination treatment in the MIA PaCa‐2 tumor mouse model of no treatment, EC‐EtOH, PDT (BPD + light), and BPD‐EC‐EtOH + light groups. Figure S5. Normalized individual animal tumor burden for multicycle combination treatment in the HepG2 tumor mouse model of no treatment, EC‐EtOH, PDT (BPD + light), and BPD‐EC‐EtOH + light groups. [file BTM2-10-e70028-s001.docx]

**Figure S1.** Layout and details for the analysis of SOSG data.

Following the injection of BPD-EC-EtOH into the phantoms, the front section of the phantoms was exposed. Subsequently, five 5-mm-thick biopsies were extracted using a hollow tool. These biopsies were labeled 1 to 5, with biopsy number 2 always placed at the injection site (A). The biopsies were then arranged accordingly in a 96-well plate for subsequent BPD photobleaching and SOSG detection. Each biopsy was assessed at 13 specified points (B) using a plate reader.


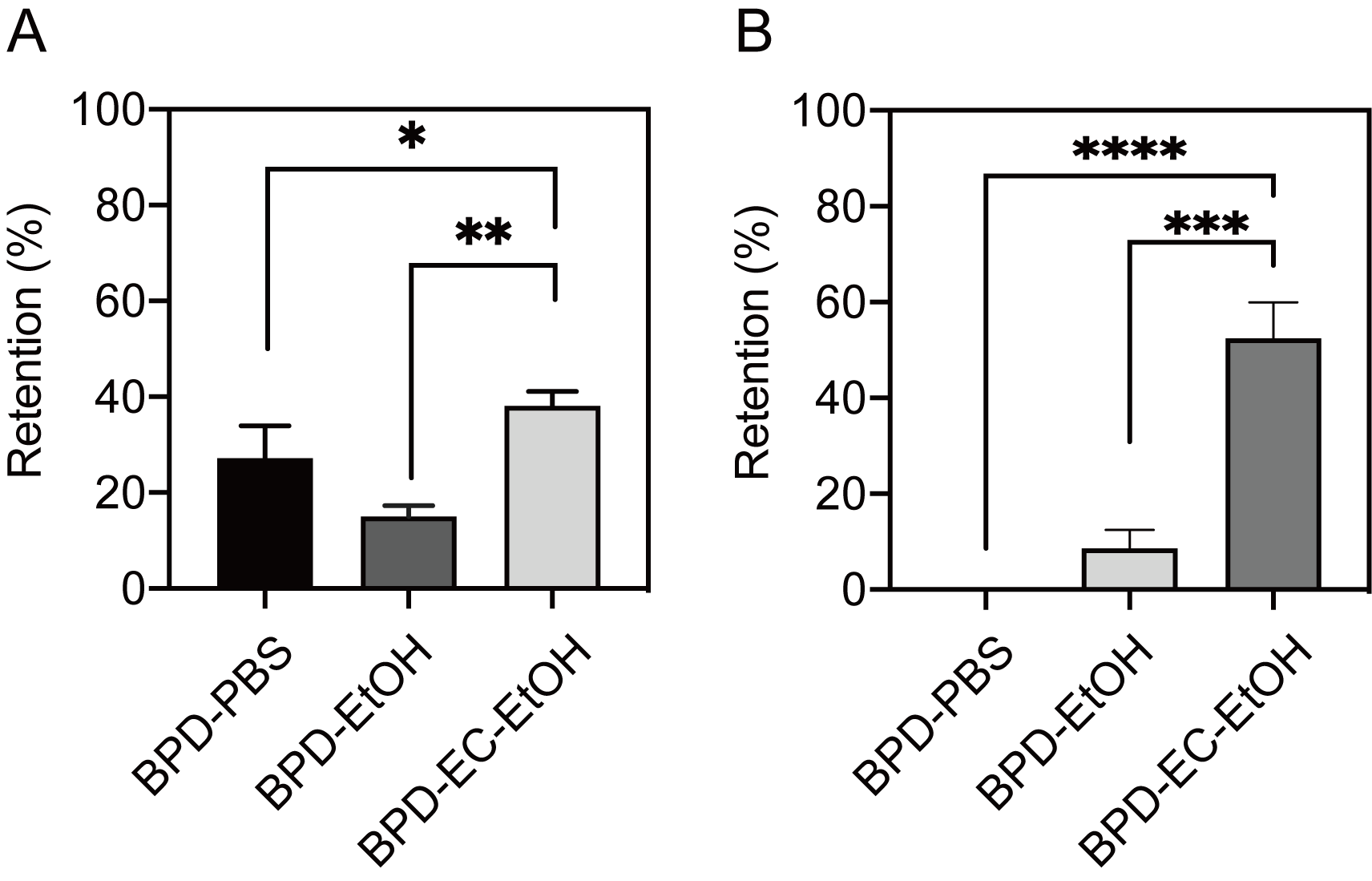


**Figure S2**. Retention rate of BPD-EC-EtOH, BPD-PBS, and BPD-EtOH in phantom (A) and swine liver (B).


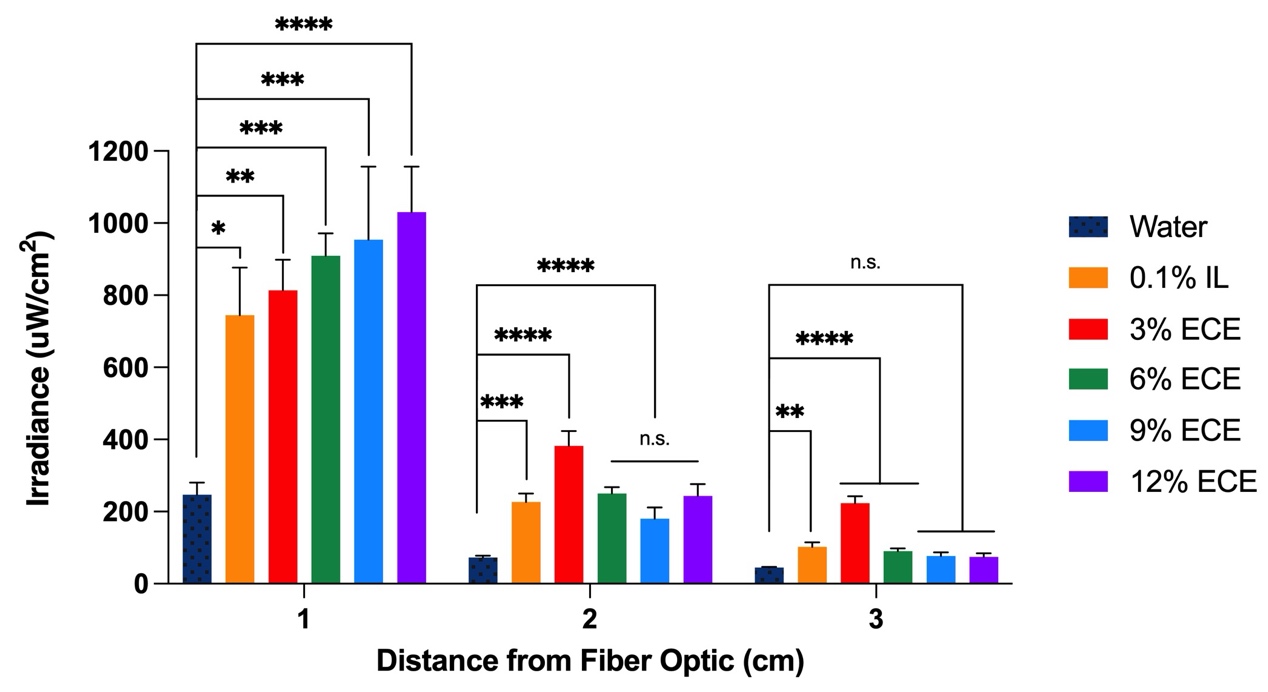


**Figure S3.** Light propagation study of 3%, 6%, 9%, 12% of EC-EtOH in pipettes.


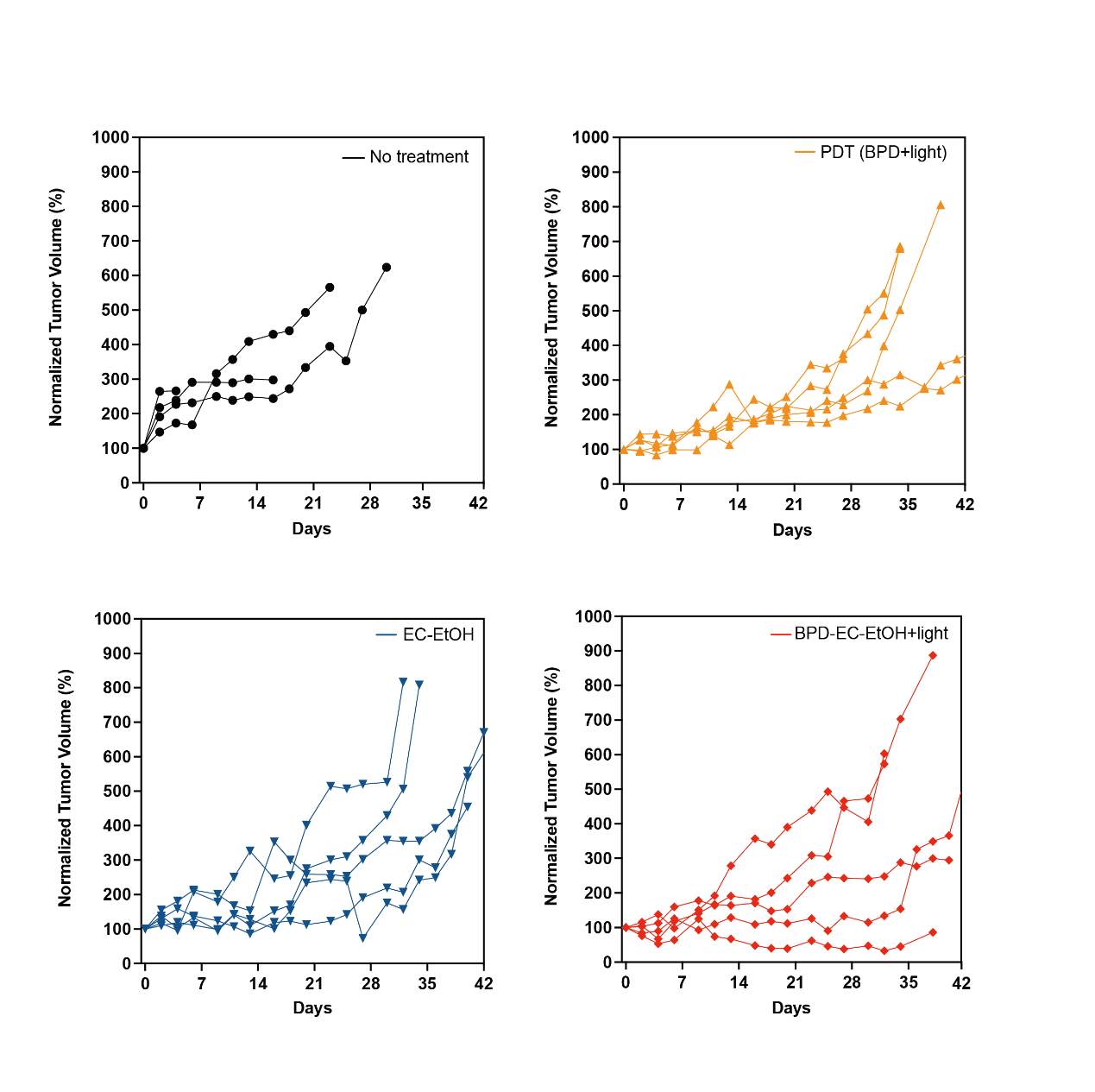


**Figure S4.** Normalized individual animal tumor burden for multicycle combination treatment in the MIA PaCa-2 tumor mouse model of no treatment, EC-EtOH, PDT (BPD + light), and BPD-EC-EtOH + light groups.


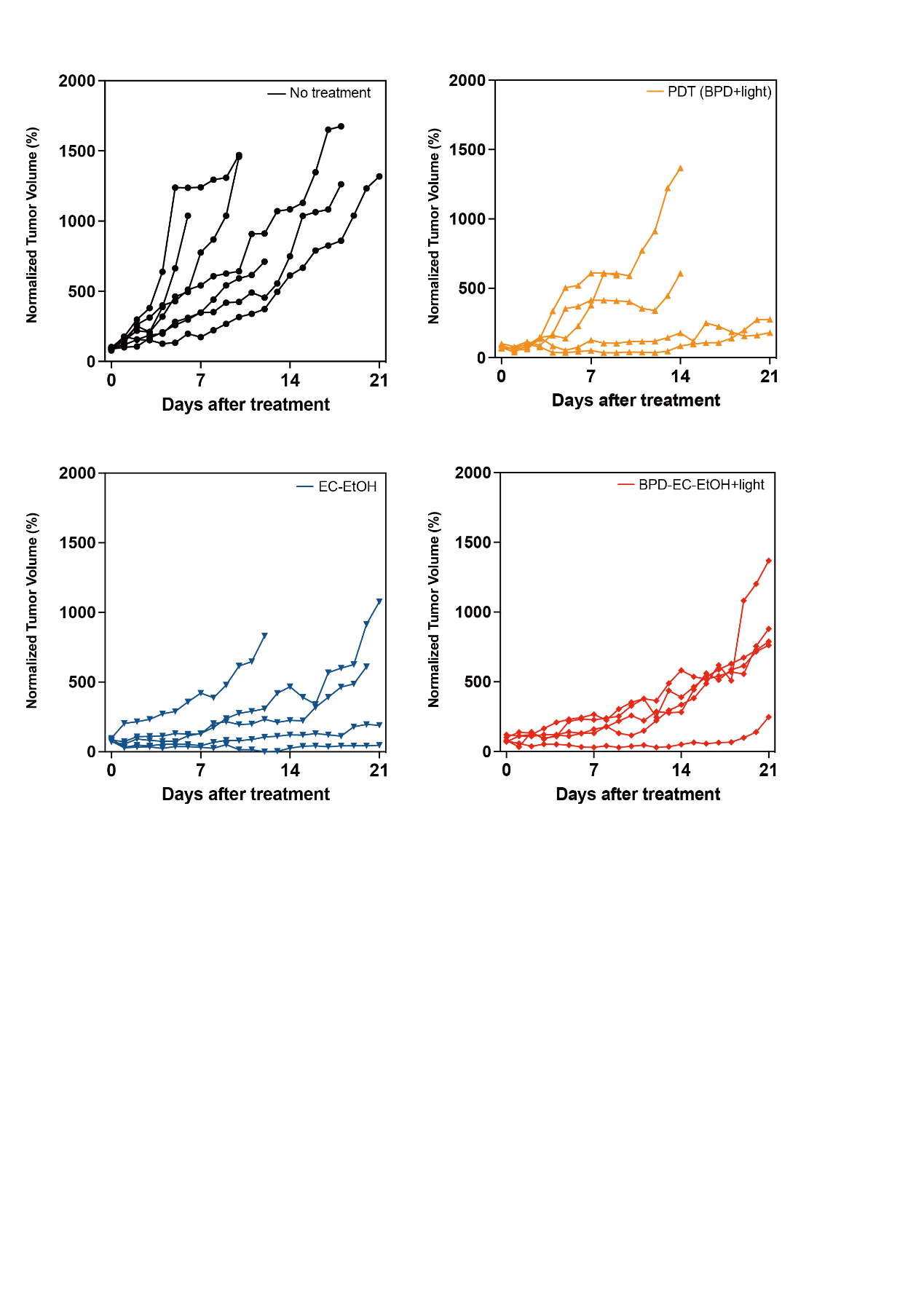


**Figure S5.** Normalized individual animal tumor burden for multicycle combination treatment in the HepG2 tumor mouse model of no treatment, EC-EtOH, PDT (BPD + light), and BPD-EC-EtOH + light groups.
